# Integrating Artificial Intelligence with Emerging Pharmaceutical Technologies: Current Progress, Clinical Translation, and Future Challenges

**DOI:** 10.3390/ijms27177864

**Published:** 2026-09-02

**Authors:** Priya Sharma, Saurabh Tiwari, Nokeun Park, Łukasz Szeleszczuk

**Affiliations:** 1 School of Pharmacy and Emerging Sciences, Baddi University of Emerging Sciences & Technology, Solan 173205, Himachal Pradesh, India; bhpriya02@gmail.com; 2 School of Materials Science and Engineering, Yeungnam University, Gyeongsan 38541, Republic of Korea; 3 Department of Organic and Physical Chemistry, Medical University of Warsaw, 1 Banacha Str., 02-097 Warsaw, Poland; lukasz.szeleszczuk@wum.edu.pl

**Keywords:** artificial intelligence in drug discovery, machine learning, nanotechnology, personalized medicine, organ-on-chip, 3D bioprinting, pharmacogenomics, drug delivery systems

## Abstract

Modern scientific and technological developments are driving major advances in drug research and development. This narrative review, based on a structured search of PubMed, Scopus, and Web of Science (2018–2026), examines how artificial intelligence (AI) and machine learning (ML) are accelerating a historically prolonged and expensive process, alongside pharmacogenomics, organ-on-a-chip systems, three-dimensional (3D) bioprinting, and nanotechnology. In benchmark studies, deep learning techniques have achieved an area under the receiver operating characteristic curve (AUROC) of over 0.85 for a subset of absorption, distribution, metabolism, excretion, and toxicity (ADMET) endpoints. AI-powered models show promising, albeit platform-dependent, accuracy in predicting candidate drug properties. Pharmacogenomics enables personalized medicine by tailoring therapies according to patients’ genetic profiles, whereas organ-on-a-chip systems and 3D bioprinting provide physiologically relevant human tissue models for preclinical evaluation. In a blinded benchmark study, the Emulate Liver-Chip showed 87% sensitivity and 100% specificity for drug-induced liver injury, outperforming animal models in that specific comparison. Nanotechnology is advancing drug delivery through the use of nanoparticle systems, such as Doxil^®^ and Onpattro^®^. Obstacles remain, including regulatory constraints, ethical considerations, data quality limitations, and the need for stronger validation, although ongoing funding, interdisciplinary collaboration, and evolving regulatory frameworks may support further development. Overall, these technologies show meaningful potential to shorten development time and improve treatment safety, although further prospective validation is required before realizing this potential at scale.

## 1. Introduction

Translational science is mainly supported by drug discovery and development, which have propelled human health advancements in a broad range of diseases [1]. These include the identification and validation of targets, optimization of hits to leads, optimization of leads, preclinical competition, and multiphase clinical testing. The entire development process takes 10–15 years on average until regulatory approval and incurs a pre-tax cost of approximately $2.6 billion per approved drug [2,3]. These timelines and costs differ significantly according to the disease area, drug class, and regulatory pathways. Priority review designations for urgent unmet medical needs may be used to shorten timelines, whereas complex chronic disease therapies are more likely to be subjected to long review periods [4]. The current situation in the pharmaceutical market provides a strong argument for radical shifts. In spite of the fact that traditional virtual screening (VS) and molecular docking are considered to be fundamental computational methods, they are limited in their accuracy and scalability. There is a growing interest in new self-reliant AI-based methods that can systematically remedy the shortcomings of traditional computational methods [5,6]. Deep learning (DL) and ML can be regarded as recent AI toolkits that demonstrate significant potential for improving drug design and discovery, although issues of interpretability and data quality remain challenging [7].

**Figure 1:**
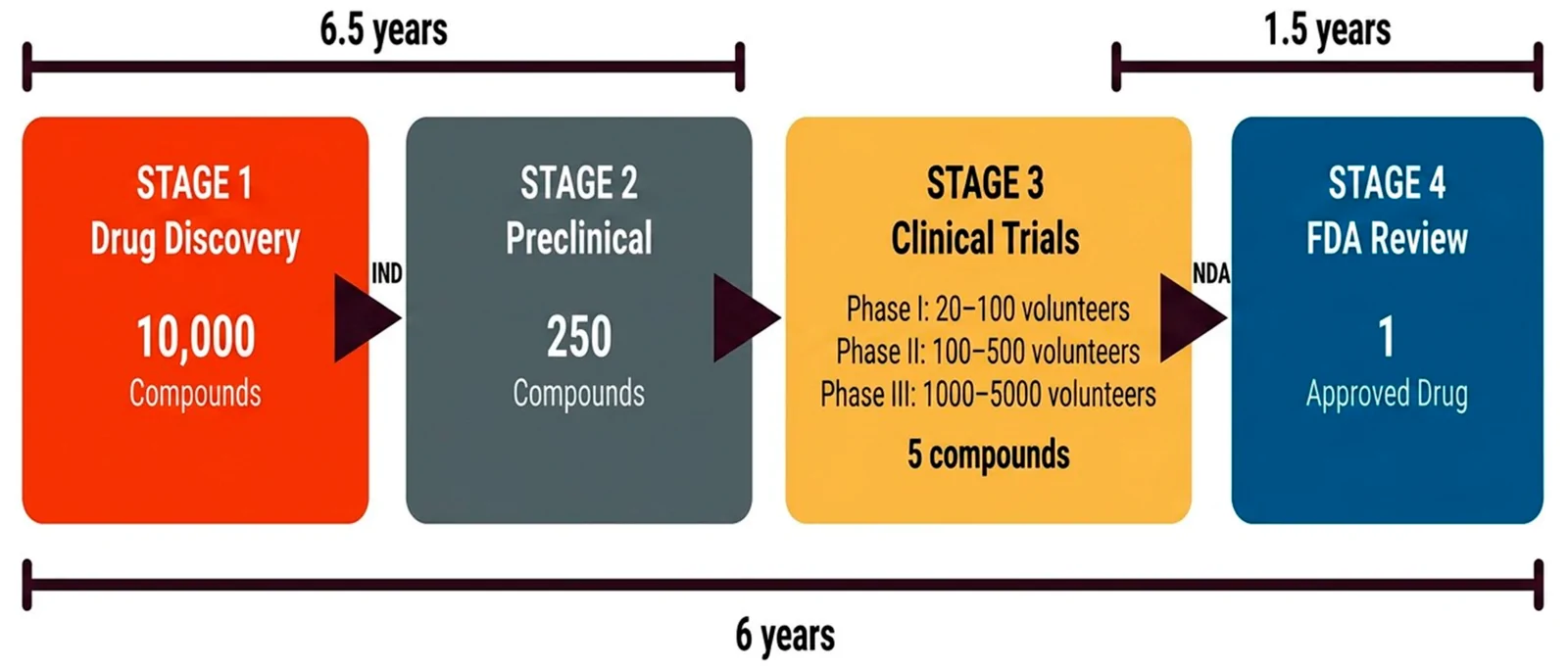
Traditional drug development process. The sequential four-stage pipeline showing Stage 1 (Drug Discovery: ~10,000 compounds, 6.5 years), Stage 2 (Preclinical: 250 compounds), Stage 3 (Clinical Trials: Phases I–III, 6 years), and Stage 4 (FDA Review: 1 FDA-approved drug, 1.5 years), with IND and NDA submission milestones.

The conventional drug development environment is well documented; however, the accelerated convergence of disruptive technologies represents a new paradigm. This narrative review summarizes recent advances in AI/ML, 3D bioprinting, organ-on-chip systems, nanotechnology, and personalized medicine, and how these areas can solve the fundamental issues of cost, time, and efficacy in the current drug development pipeline [5,8,9,10,11,12,13]. Figure 1 summarizes the conventional drug discovery and development processes, which are time-consuming and multi-stage [1]. Many reviews have discussed artificial intelligence, pharmacogenomics, organ-on-chip systems, nanotechnology, and 3D bioprinting in the context of pharmaceutical research; however, few have discussed how they all work together in a single translational model. To date, reviews conducted so far have mostly been descriptive or promotional, without thoroughly comparing the level of readiness, regulatory maturity, scalability, and failure modes of the technologies. This review adopts an integrative comparative approach, examining AI/ML, pharmacogenomics, organ-on-chip systems, nanotechnology, and 3D bioprinting using a common set of translational criteria, including technological maturity, clinical readiness, regulatory considerations, and limitations, rather than presenting them as a fully unified translational model. We compared the levels of maturity and translational potential and highlighted the possibilities and problems that remain unresolved. By comparing these technologies side by side, this review addresses a specific gap in the existing literature: the lack of a side-by-side comparison of the translational readiness, regulatory maturity, scalability, and key limitations of these five technology domains within a common framework. It also highlights the methodological, ethical, and regulatory issues that need to be addressed for these technologies to have an effect in the real world. This review critically compares the translational readiness of AI/ML, pharmacogenomics, organ-on-chip systems, three-dimensional bioprinting, and nanotechnology in drug discovery and identifies where their integration offers the greatest potential to improve efficiency, reduce development time and cost, and facilitate clinical translation.

## 2. Scope and Literature Selection

This narrative review employed a structured literature search strategy to ensure transparency in the identification and selection of the relevant literature. Relevant publications were identified through searches of PubMed, Scopus, and Web of Science, primarily covering 2018–2026 to capture recent advances in artificial intelligence, pharmacogenomics, organ-on-chip systems, nanotechnology and three-dimensional bioprinting. Pre-2018 landmark studies were also included, as they provided foundational evidence for specific technologies, including early organ-on-chip validation studies and CPIC pharmacogenomic guidelines. The search terms included combinations of the following: AI drug discovery, machine learning pharmacogenomics, organ-on-chip drug testing, nanomedicine delivery, and 3D bioprinting in pharmaceuticals. The inclusion criteria were peer-reviewed articles, regulatory reports, and case studies that presented empirical data or critical analyses of translational preparedness. Editorials lacking peer review, promotional white papers, and studies with inadequate methodological transparency were excluded from the study. The reference lists of the key articles were also examined to identify additional relevant sources. Priority was given to studies reporting quantifiable outcomes (e.g., development timelines, sensitivity/specificity, and cost implications) and those addressing the limitations or regulatory considerations. This strategy enabled the integration of high-impact case studies with a broader scholarly perspective while minimizing over-generalization and unsupported assertions. A total of 80 publications and relevant sources were incorporated into this narrative review, including peer-reviewed articles supplemented by guidelines, regulatory documents, industry sources, and book chapters.

## 3. Role of Artificial Intelligence and Machine Learning in Drug Development

AI allows the creation of applications that can simulate human cognitive behaviors. In the AI paradigm, ML is a unique paradigm that enables algorithms to process data and generate intelligent behavioral patterns without explicit programming [5]. ML encompasses DL as a specialized subdomain of application that uses neural network architectures based on the brain structure theory. The development of AI has taken two axes: accurate simulation of human cognition and rational decision-making systems that can make logical inferences. The area of AI research includes the general and specialized areas of reasoning, learning, perception, game playing, and natural language processing [14].

**Figure 2:**
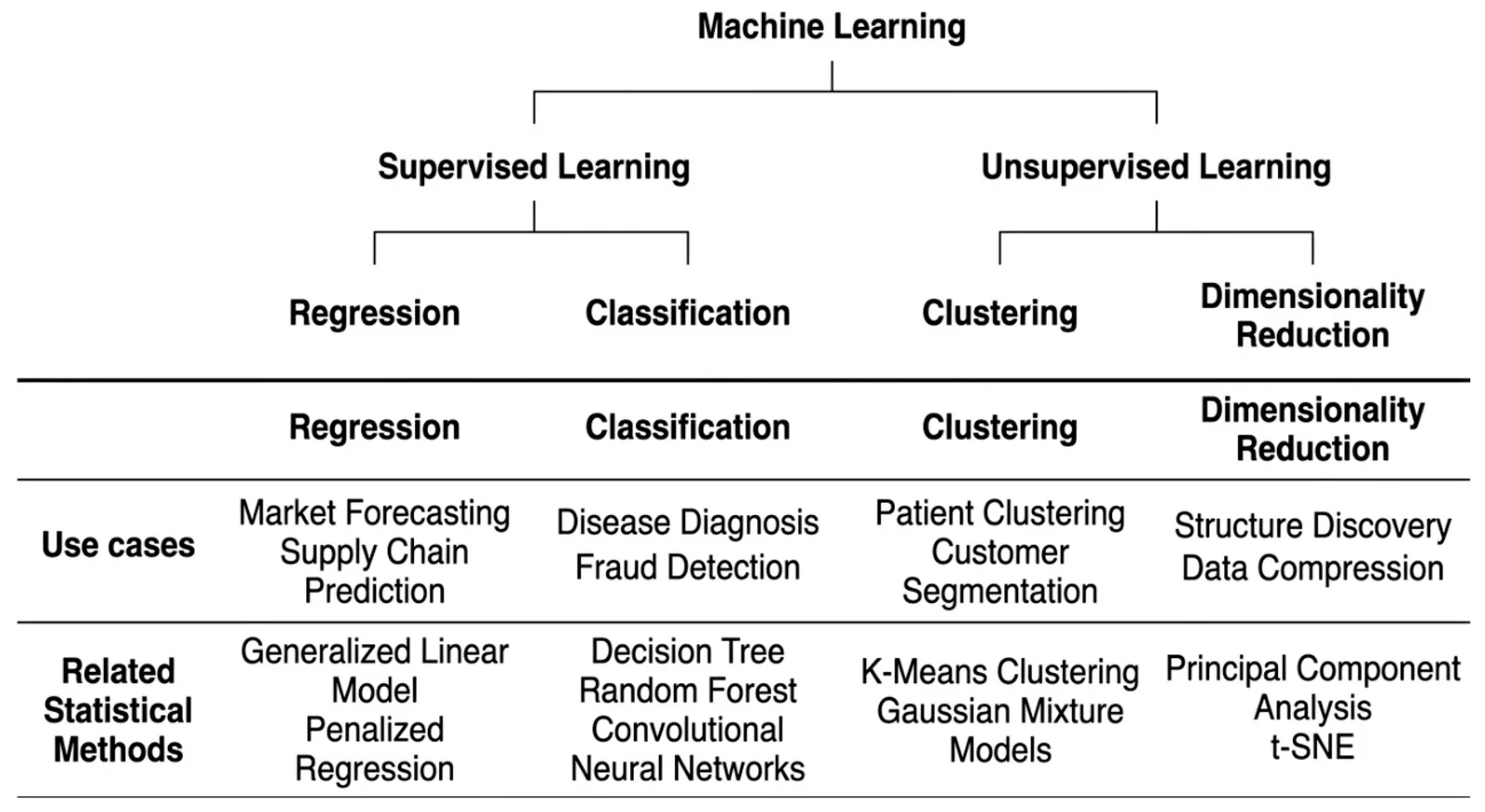
Machine learning-related statistical methods. Classification of essential ML strategies (Supervised Learning: regression and classification; Unsupervised Learning: clustering and dimensionality reduction) and their applications and related statistical tools applicable to pharmaceutical research studies.

AI technology is based on the fields of philosophy, mathematics, statistics, economics, neuroscience, psychology, computer engineering, control theory, and linguistics. Within the ML paradigm, various method-related algorithms can assist researchers in identifying patterns in complex datasets. ML can be divided into three major categories: supervised learning, unsupervised learning, and reinforcement learning, which are supported by specific statistical tools that are crucial in the data analysis of drug discovery [5,7]. The hierarchical relationship between AI, ML, and DL is as follows: AI is the broadest field, ML is a subset of AI that enables learning from data without explicit programming, and DL is a specialized subdomain of ML that uses neural network architectures that are inspired by biological neural networks. Figure 2 classifies the core ML approaches and their pharmaceutical applications [5].

There is documented evidence that AI-accelerated programs demonstrate substantially reduced time to clinical candidate nomination: DSP-1181 reached clinical candidacy in less than 12 months compared with the industry average of 4.5 years [15], and AI-accelerated virtual screening of over 100 million molecules discovered the novel antibiotic halicin in days rather than years [16]. Such gains can be explained by the ability of AI to combine multi-source and multi-scale biological information (genomics, proteomics, chemical structure, and clinical outcomes) into a single predictive model. However, not all AI-designed candidates progress clinically; for example, DSP-1181 was discontinued in 2022 without advancing to Phase II, illustrating that AI-accelerated candidate nomination does not guarantee downstream clinical success [17]. In contrast, rentosertib (ISM001-055) became the first AI-designed drug to demonstrate clinical proof of concept in a Phase IIa randomized trial, with published results in 2025 confirming improved lung function in patients with IPF [18,19]. The leading AI discovery platforms by 2025 include generative chemistry, phenomics-first systems, integrated target-to-design pipelines, knowledge-graph repurposing, and physics-plus-ML design, each demonstrating distinct strengths and failure modes. However, no single platform has achieved consistent clinical translation [20]. Several structural factors help explain why AI-nominated candidates frequently fail to progress, despite strong in silico performance. First, most nomination models are trained and validated against biochemical or cellular endpoints (binding affinity, in vitro ADMET), which are imperfect surrogates for in vivo efficacy, systemic toxicity, and immunogenicity properties that only emerge in later-stage testing. Second, models trained on historically available, chemically biased datasets can perform poorly when applied to genuinely novel chemotypes, which is a distributional-shift problem that is difficult to detect prior to synthesis. Third, the case of DSP-1181 illustrates that rapid, AI-driven candidate nomination does not by itself de-risk the later, more expensive stages of clinical development, where attrition is driven by the same biological and regulatory factors that limit conventional drug candidates [4,17]. The public record for DSP-1181 discontinuation does not disclose a specific mechanistic reason, and we do not speculate beyond what is reported.

### 3.1. Artificial Neural Networks

Artificial neural networks (ANNs) are computational functions inspired by biological neural networks [21]. The basic ANN is a fully connected feedforward network that consists of input, hidden, and output layers. Each layer has neurons that perform nonlinear mathematical operations on the input data they receive, and information flows in a dotted manner from one layer to the next. ANNs are highly effective in pharmaceutical research because they can identify complex, nonlinear relationships [5].

ANNs are used in critical processes of molecular modeling and drug development to solve complicated problems in High-Throughput Virtual Screening (HTVS) and Quantitative Structure–Activity Relationship (QSAR) analysis, along with pharmacokinetic and pharmacodynamic modeling.

**Figure 3:**
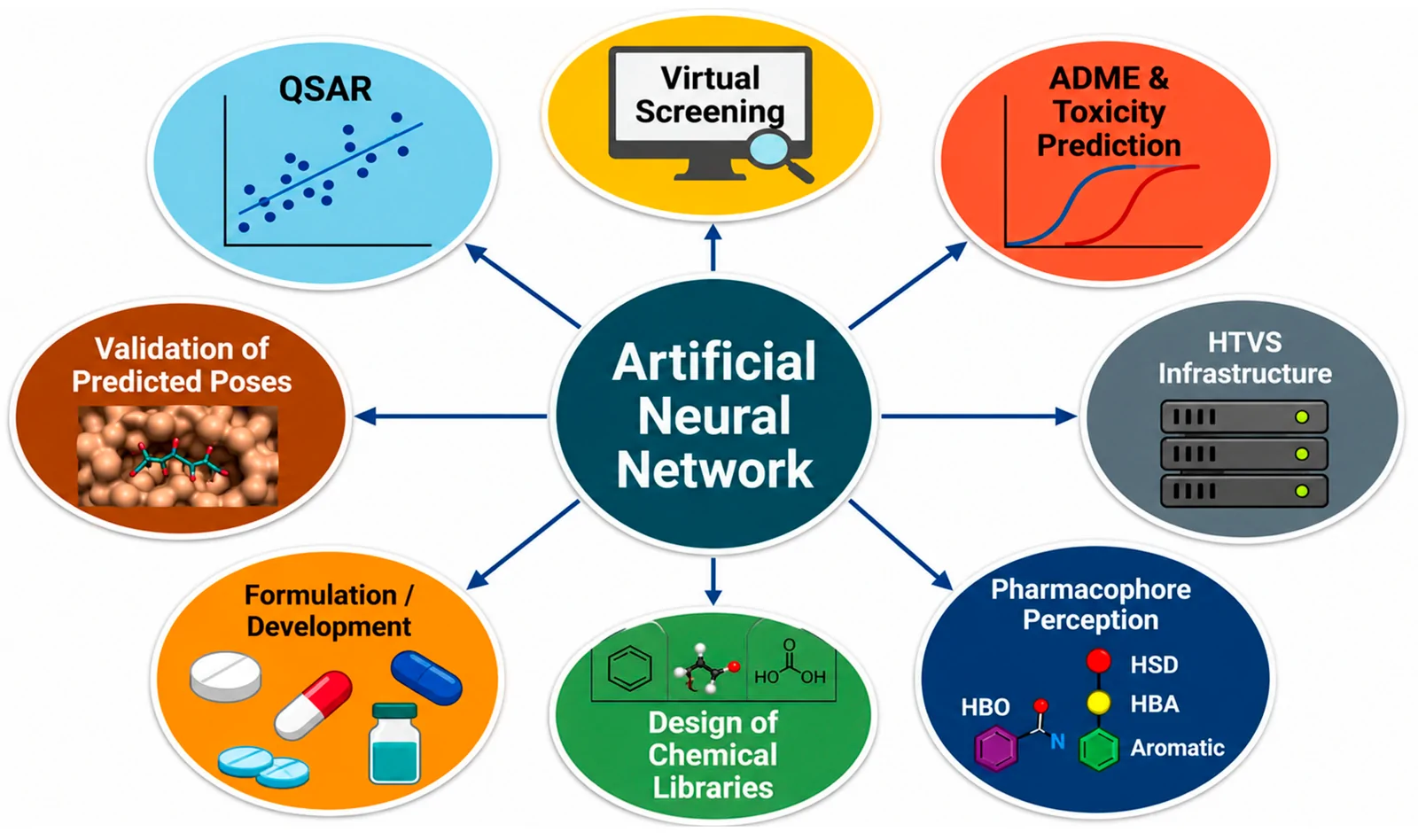
Application of artificial neural networks in drug discovery. An illustration of ANN applications.

ANNs can be used to predict the in vivo absorption, distribution, metabolism, and excretion (ADME) of a new drug candidate or screen billions of compounds in silico to shortlist the most promising compounds for laboratory testing, thus dramatically accelerating the early drug discovery phase. Deep learning models fed with large chemical collections have achieved an AUROC > 0.85 for 20 of 31 classification datasets and an R^2^ > 0.6 for 5 of 10 regression datasets in a large multi-task ADMET benchmark, representing an improvement over several previous QSAR approaches for the endpoints where this threshold was met, rather than for ADMET prediction in general [22]. Figure 3 illustrates the application of ANNs in predicting drug-target interactions and ADMET properties [5,22]. Despite these applications, ANN-based approaches face specific limitations in pharmaceutical applications. They generally require larger, better-curated datasets than those typically available for a given target class or chemical series, and their predictive accuracy may decrease outside the chemical space represented in the training data. The resulting interpretability gap, the difficulty in explaining why a specific compound was flagged, remains a practical barrier to regulatory acceptance and medicinal chemists’ trust in model output [23].

### 3.2. Drug Repurposing

The predictive and pattern recognition capabilities of AI and ML have enabled remarkable progress in drug repurposing (also known as repositioning). This novel approach reveals new therapeutic applications for existing medications, including those that are approved, archived, or undergoing clinical trials to treat various emerging diseases [24]. AI algorithms can process large volumes of clinical records, genomic data, and drug-related data to identify potential drug–disease associations that are difficult to detect by manual analysis. Because repurposed drugs have typically already undergone clinical safety testing for their original indication, AI-assisted repurposing can shorten some early development steps, providing a comparatively quick and cost-effective route relative to novel compound development, although clinical validation and regulatory evaluation for the new indication remain necessary [24].

#### 3.2.1. Case Study 1: Baricitinib for COVID-19 (BenevolentAI)

The coronavirus disease (COVID-19) pandemic has demonstrated the potential of AI-driven drug repurposing. In February 2020, an AI system developed by BenevolentAI, a knowledge graph-based approach to AI, found a COVID-19 therapeutic in baricitinib, an established JAK inhibitor used to treat rheumatoid arthritis, by analyzing its capacity to decrease viral entry through AAK1 inhibition and counteract the resultant cytokine-mediated inflammatory response [25]. The AI analysis took a few days to develop a hypothesis that could be tested. Later preclinical validation was able to validate the prediction, with clinical trials showing efficacy in hospitalized patients, resulting in FDA Emergency Use Authorization in November 2020 and full approval in combination with remdesivir in 2022 [25,26]. This is an approximate 9-month period of AI prediction to regulatory approval, versus the normal 10–15 years of development of novel drugs [25,26]. Notably, this compressed timeline reflects the combination of a rapid AI-generated hypothesis with an already approved, well-characterized drug and pandemic-era regulatory fast-tracking; the 9-month interval is therefore not representative of the timeline achievable for a novel, AI-designed compound without an existing safety record. Since then, hundreds of thousands of hospitalized COVID-19 patients around the globe have been treated with baricitinib, confirming the revolutionary nature of AI-mediated repurposing as a rapid therapeutic response [27].

#### 3.2.2. Case Study 2: DSP-1181—First AI-Designed Drug to Enter Clinical Trials (Exscientia/Sumitomo Dainippon)

A landmark example of AI-accelerated drug design is DSP-1181, an obsessive–compulsive disorder (OCD) treatment developed by Exscientia in collaboration with Sumitomo Dainippon Pharma. This molecule was the first AI-designed drug to enter clinical trials in 2020 [15,28]. Using the AI-driven design process, it only required 12 months to nominate a clinical candidate after project initiation, as compared to the 4–5 years needed when incorporating conventional methods [15,28]. Using its AI platform, Exscientia generated 350 candidate compounds, which were synthesized and experimentally tested to identify the optimal clinical candidate [15,28].

Compared with conventional drug discovery programs requiring substantially larger numbers of synthesized compounds, this productivity demonstrates how AI can significantly decrease time and resource consumption. In January 2020, DSP-1181 became the first AI-driven drug design to enter Phase I trials in Japan, demonstrating the feasibility and efficiency of AI-driven drug design.

**Figure 4:**
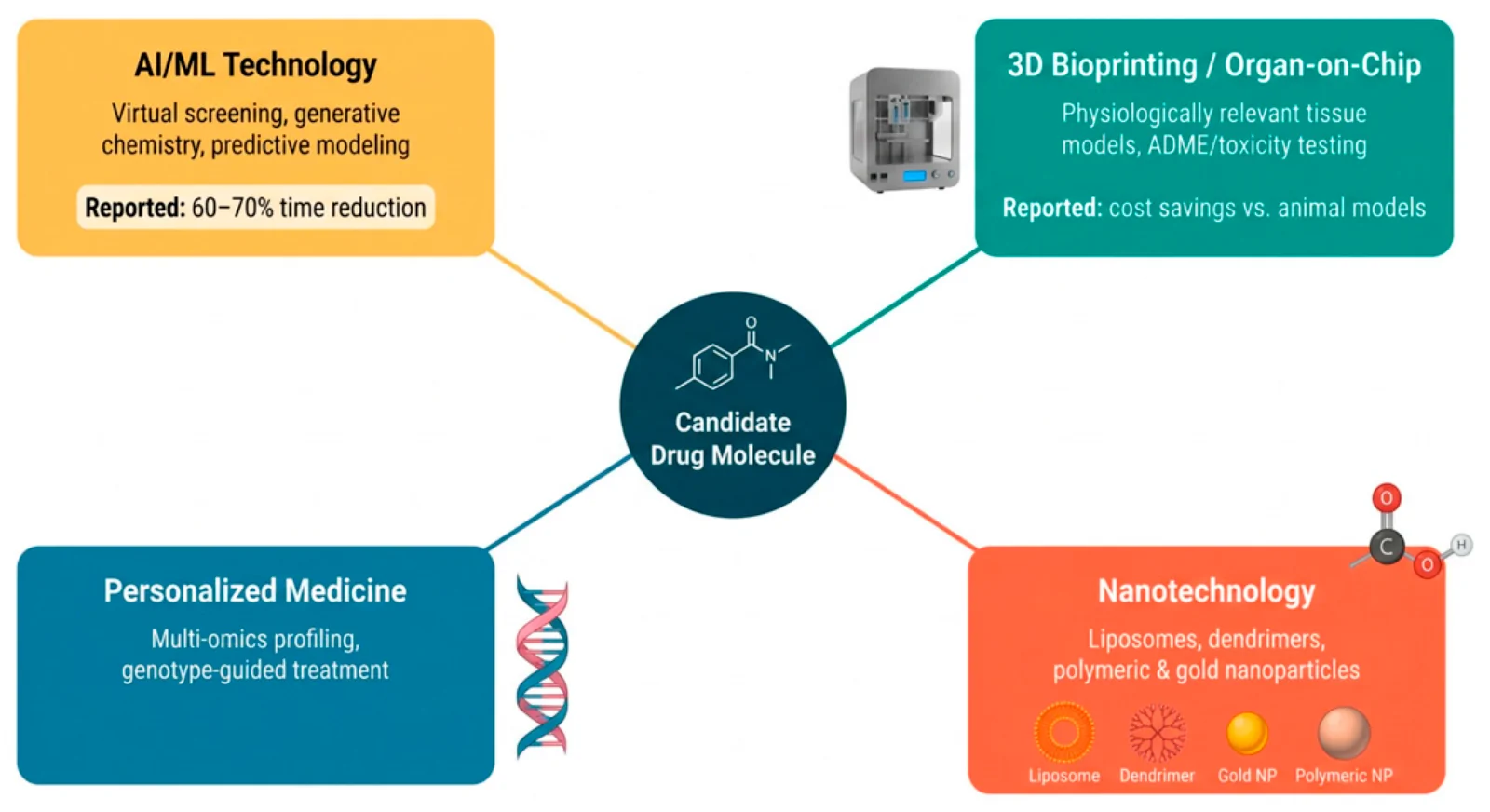
Integrated drug discovery technology ecosystem, illustrating how AI/ML technology, personalized medicine (genotype-guided treatment), organ-on-chip and 3D bioprinting systems, and nanotechnology converge around a candidate drug molecule. The representative benefits are based on selected AI-enabled pharmaceutical workflows and are not universal benchmarks.

**Figure 5:**
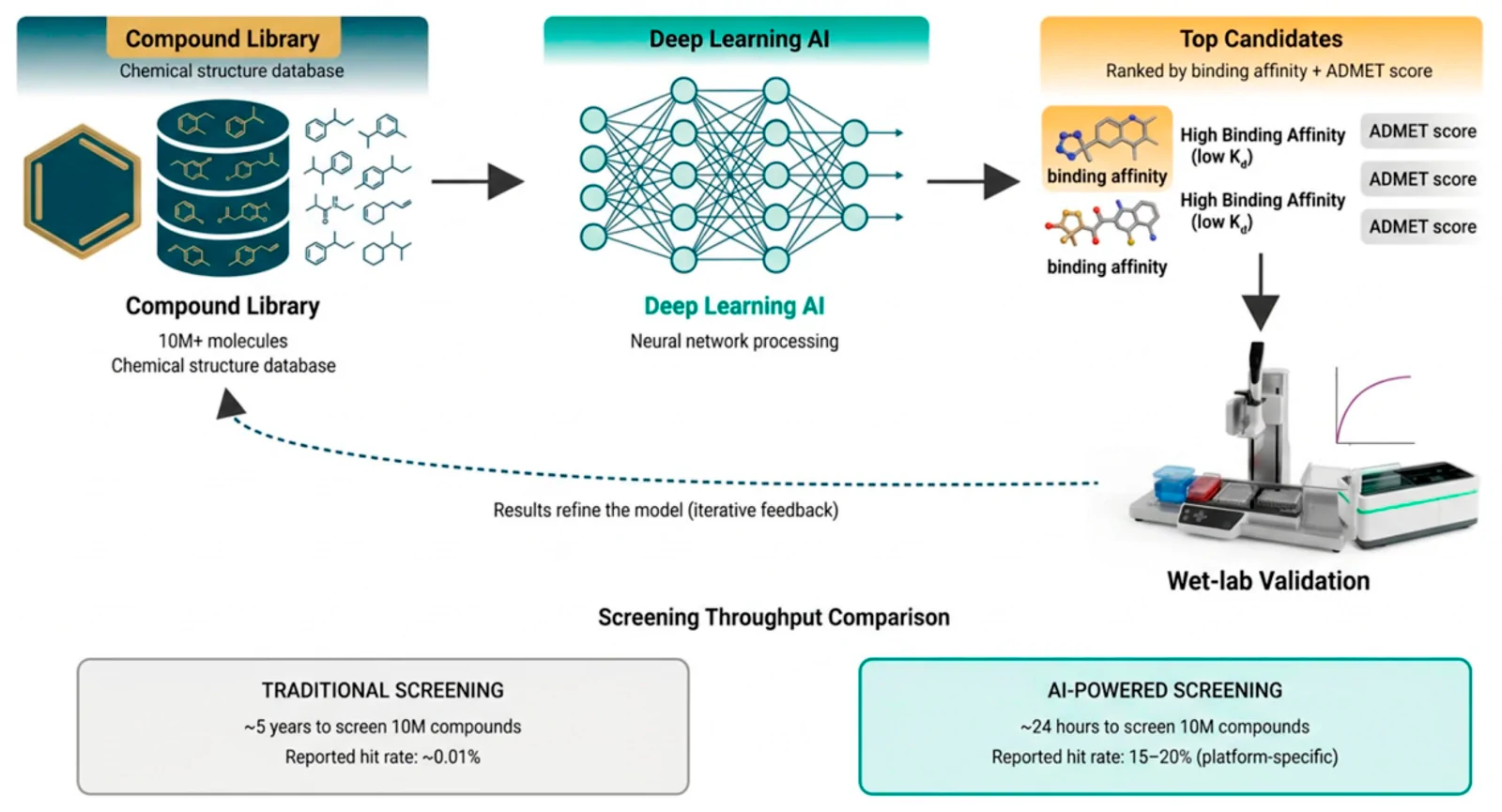
AI-Powered Virtual Screening Pipeline for Drug Discovery. The percentage figures shown in the figure are illustrative of platform-reported estimates and should not be interpreted as universally validated benchmarks.

Figure 4 (Integrated Drug Discovery Technology Ecosystem) offers a generalized visualization of how AI/ML, 3D bioprinting, nanotechnology, and personalized medicine are applied to a central drug molecule, and some of the key metrics illustrate their combined effect. Figure 5 (AI-Powered Virtual Screening Pipeline) shows the workflow in a 10M+ compound library from deep learning neural network processing to top drug candidates with binding affinity and ADMET scores, with hit rates that are significantly better compared to traditional screening, as exemplified by the discovery of halicin out of >100 million compounds [16], and the capacity to screen 10 million compounds in 24 h versus 5 years for conventional approaches.

## 4. Personalized Medicine and Pharmacogenomics

Pharmacogenomics is a new technology that combines the knowledge of genomics with the use of drugs to determine the treatment programs that a patient would receive, which has revolutionized precision healthcare. Pharmacogenomics is a step beyond the traditional one-size-fits-all medicine prescriptions, using personalized medication selections that consider each patient with a unique genetic profile, improving the effectiveness of the drug and minimizing adverse effects [29]. The completion of the Human Genome Project provided background information on drug-metabolizing enzyme polymorphisms, especially those of the cytochrome P450 (CYP450) family, which significantly influences drug metabolism [29]. Differences in drug processing speeds caused by CYP450 variants result in clinically significant changes in drug efficacy and toxicity [29].

**Figure 6:**
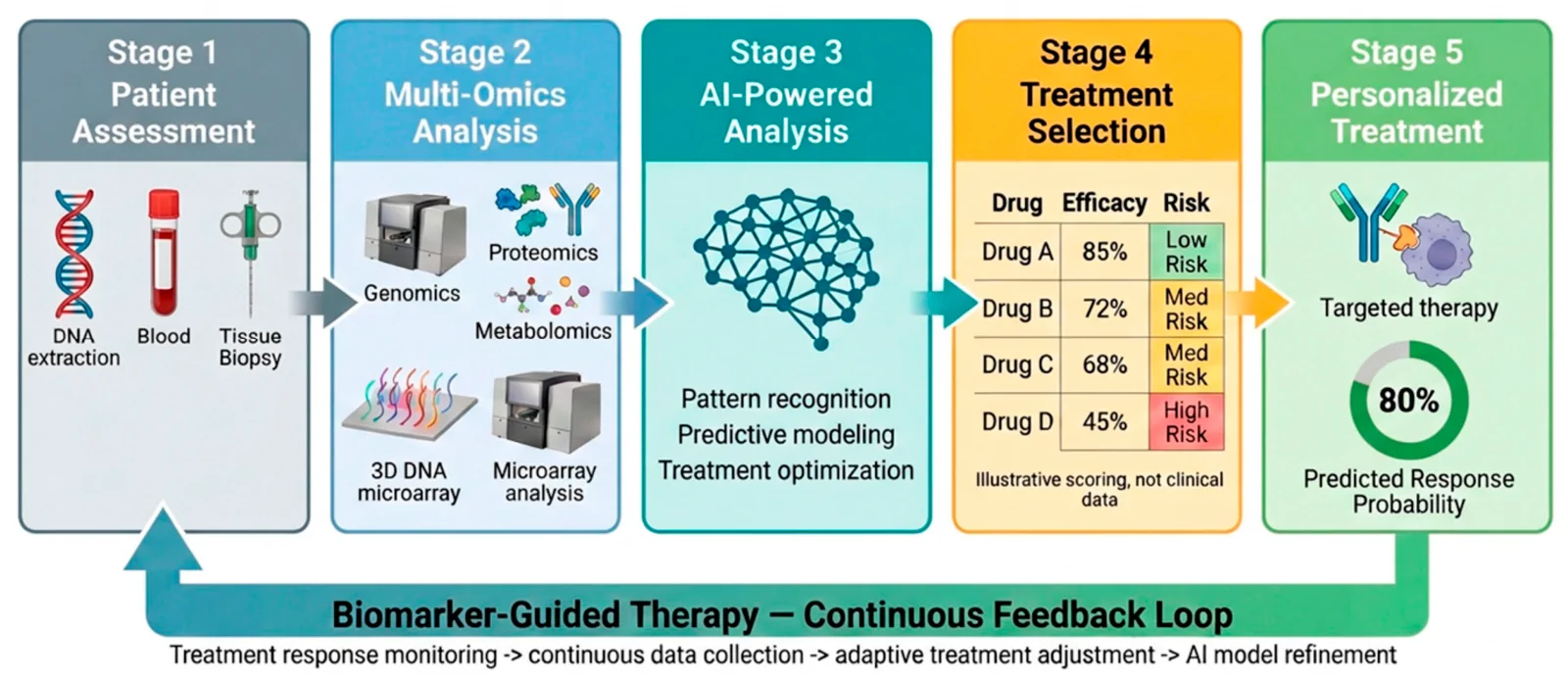
A personalized medicine clinical decision pathway integrates patient assessment, multi-omics analysis, AI-based prediction, treatment selection, and personalized therapy. The feedback loop represents continuous response monitoring, data collection, treatment adjustment and AI model refinement. Stage 4 efficacy scores are illustrative values for a hypothetical decision tree and do not represent the results of a specific clinical trial.

Drug therapy outcomes reach their optimal level when pharmacogenomics connects treatment decisions to an individual’s genetic profile. Genetic variations in drug transporters, targets, receptors, and absorption and distribution mechanisms collectively determine the magnitude of the pharmacological response in patients [29,30]. The evolving discipline of pharmacogenomics has the potential to transform patient-focused medical care and redefine contemporary healthcare and wellness paradigms. Academia and healthcare practices must increasingly adopt pharmacogenomics as an essential component for creating effective and equitable healthcare [31]. Figure 6 (Personalized Medicine Clinical Decision Pathway) illustrates the five integrated stages [29,32] of the precision medicine workflow: patient assessment, multi-omics analysis, AI-powered analysis, treatment selection with predicted efficacy scores, and personalized treatment with continuous biomarker-guided monitoring, embodying the precision medicine principle: ‘Right Drug, Right Patient, Right Time.’

### 4.1. Genetic Profiling

Individuals process drugs differently because of their genetic compositions. CYP450 enzyme polymorphisms create a spectrum of metabolizer phenotypes (poor, intermediate, extensive, and ultra-rapid), which directly translate into variations in drug plasma concentrations, therapeutic efficacy, and adverse effect profiles of drugs. Genetic variations in drug transporters (e.g., *SLCO1B1* for statin-related myopathy), receptors (e.g., OPRM1 for opioid response), and targets further determine the pharmacological responses in patients. The application of genetic knowledge allows clinicians to select drugs and adjust drug dosages to minimize adverse drug reactions (ADRs), which drives personalized and more effective pharmacotherapy [29,30].

### 4.2. Clinical Applications of Pharmacogenomics

Clinical pharmacogenomics is increasingly being incorporated into several therapeutic areas to support evidence-based genotype-guided treatment. In parallel with the Clinical Pharmacogenetics Implementation Consortium (CPIC) in the United States [33], the Dutch Pharmacogenetics Working Group (DPWG) provides gene–drug dosing recommendations that are widely used in European clinical practice. Although the recommendations of the two organizations are largely concordant, differences exist for some gene–drug pairs and specific prescribing recommendations. In oncology, pharmacogenomic testing can guide the selection and dosing of chemotherapeutic agents. For example, *UGT1A1* genotyping can help inform irinotecan dosing, whereas *TPMT* and *NUDT15* testing can guide thiopurine therapy, including in patients with acute lymphoblastic leukemia [34]. Multi-omics and biomarker-matched approaches in precision oncology have been associated with improved objective response rates relative to non-matched or standard chemotherapy in several precision-oncology trials, although the magnitude of benefit varies considerably by trial design and tumor type [32].

Pharmacogenomics has important applications in cardiovascular medicine. *SLCO1B1* genotyping can help identify patients at an increased risk of statin-associated myopathy, whereas *CYP2C19* genotyping can inform clopidogrel therapy by identifying patients with reduced metabolic activation of the drug. Similarly, *VKORC1* and *CYP2C9* genotypes can provide information relevant to warfarin dosing [35,36]. In psychiatry, *CYP2D6* and *CYP2C19* genotyping may support the selection and dosing of antidepressants and antipsychotics, potentially reducing treatment-related adverse effects and shortening the time required to achieve an effective regimen [37]. Beyond individual gene–drug associations, pharmacogenomics is increasingly integrated with clinical and real-world patient information to support individualized treatment decisions. The combination of genomic and multi-omics profiles with clinical characteristics can help identify treatment strategies tailored to individual patients. Pharmacogenomics-informed prescribing may also improve medication management by reducing adverse drug reactions, preventing drug-related hospitalizations, and potentially lowering healthcare costs [38]. Further integration with wearable monitoring, clinical data, and AI-based decision support systems could enable continuous refinement of treatment strategies as additional patient-specific information becomes available [32]. Broader clinical implementation nonetheless remains constrained by workflow, reimbursement, and infrastructure barriers that vary across healthcare systems [39]. Table 1 summarizes the representative gene–drug pairs discussed in this section, along with their corresponding clinical recommendations.

Pharmacogenomics is the most clinically mature technology reviewed, supported by the CPIC and DPWG guidelines. However, broader implementation remains limited by genotyping costs, healthcare infrastructure, and the underrepresentation of non-European populations in the evidence base.

## 5. Three-Dimensional Bioprinting and Artificial Organ Models

Additive manufacturing (also known as three-dimensional (3D) printing) has fundamentally transformed the field of modern medicine. This technique is used to build complex structures based on computer-aided design (CAD) using materials that are deposited in a precise spatial manner. In contrast to classic subtractive production, 3D printing creates structures in an additive manner, allowing outstanding precision, product customization, and the creation of highly complex geometries. Medicine is one field where the concept of 3D printing finds many applications, such as custom prosthetics, orthopedic implants, point-of-care anatomical surgical planning models, and educational tissue phantoms [40]. The most revolutionary aspect of this technology is the production of functional human tissues and organs, commonly termed organ bioprinting [41,42]. Developers will create biological architectures that recapitulate the natural architecture and functions of human tissues. This involves the use of bioinks, which are special materials composed of living cells, growth factors, and extracellular matrix (ECM) components [43]. Bioinks are deposited layer-by-layer using bioprinters designed to work with fragile biological materials. Stem cells are the general building blocks for assembling complex tissue constructs because of their ability to differentiate into various cell types [41]. The creation of in vitro organ models using 3D bioprinting provides researchers with physiologically relevant drug evaluation systems for therapeutic research, disease modeling, and toxicological investigations, including 3D bioprinted tumor microenvironments for oncology drug testing [44]. There are two main technical methods: indirect printing, in which a sacrificial mold is created and then filled with biological material, and direct printing, in which the organ model is created on the printing platform. The most common 3D printing modalities of organ models are stereolithography (SLA), fused deposition modeling (FDM), inkjet-based 3D printing, and digital light processing (DLP), each with its own set of strengths and weaknesses [40] that are adapted to various tissue engineering applications.

### 5.1. Precision in Preclinical Testing: Organ-on-Chip Technology

**Figure 7:**
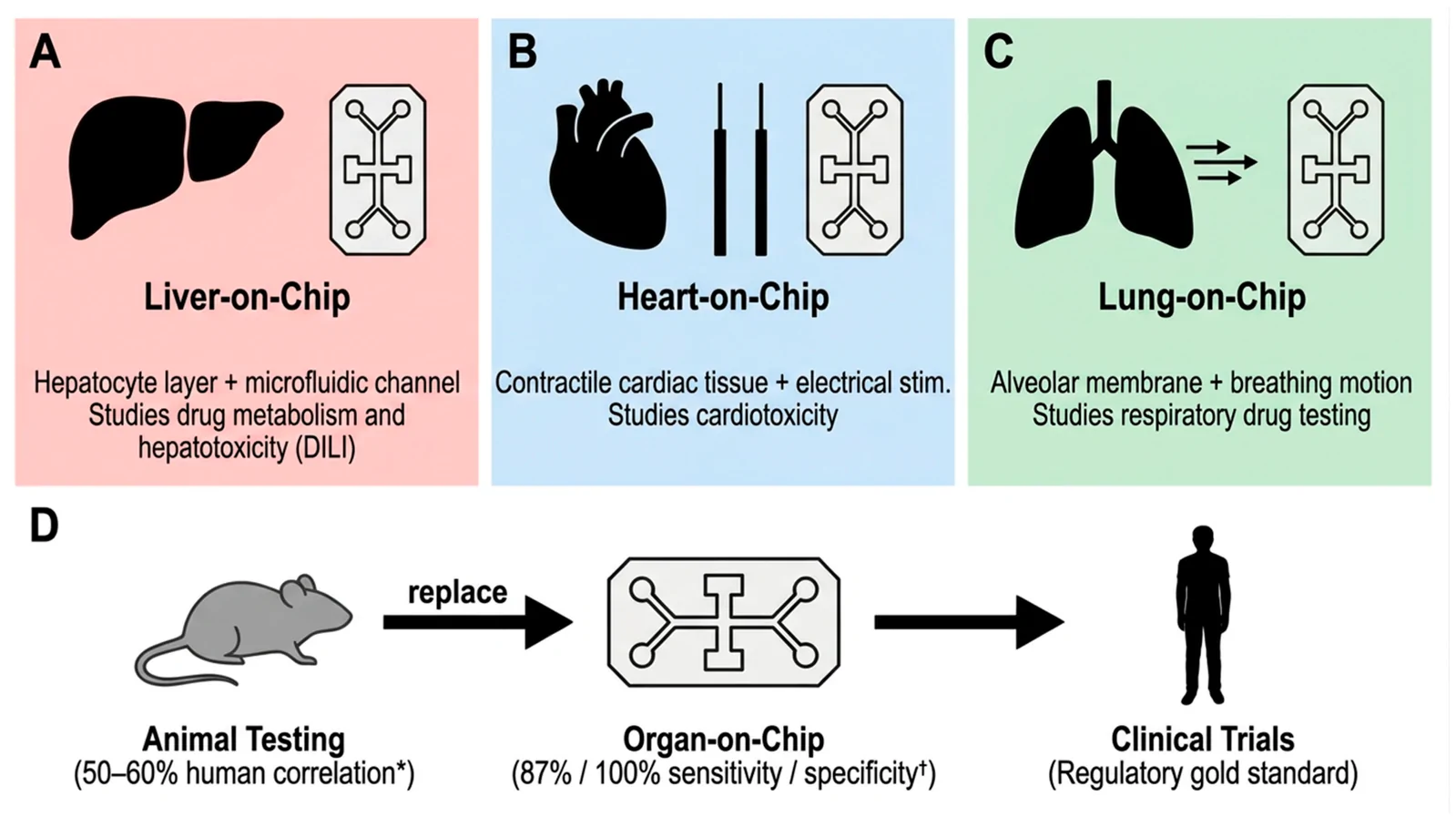
Organ-on-chip platforms for drug testing and their comparative preclinical predictive potential. (**A**) Liver-on-chip for drug metabolism and drug-induced liver injury (DILI) assessment; (**B**) heart-on-chip for cardiotoxicity testing; (**C**) lung-on-chip for respiratory drug evaluation; and (**D**) comparison of animal testing, organ-on-chip platforms, and clinical trials. * The 50–60% human-correlation estimate represents a general preclinical estimate from the broader literature and is not derived from the cited liver-chip study. † The 87% sensitivity and 100% specificity values are specific to the cited liver-chip study. Clinical trials remain the regulatory gold standard.

Organ-on-chip (OoC) technology is a fundamental innovation in drug development and testing. These microphysiological systems (MPS) recreate aspects of human organ architecture and mechanical and biochemical processes at the microscale level. Unlike organoids, which are self-organizing three-dimensional cellular constructs that recapitulate aspects of tissue architecture [45], OoC devices incorporate microfluidic channels to provide controlled perfusion, mechanical stimulation, and physiologically relevant microenvironments for the cells. By fusing tissue engineering, microfluidic design, and biomaterial science, researchers have built platforms that bridge in vitro testing and human body dynamics. OoC devices enable physiologically relevant fluid shear stress, multicellular communication, and three-dimensional (3D) biomimetic environments [46]. These models exceed conventional two-dimensional (2D) in vitro culture systems because they replicate cellular microenvironments more accurately, support correct tissue function, and better simulate human organ behavior [10]. Researchers can monitor biological functions in real time, including cellular proliferation, developmental processes, signal transduction, and metabolic activity [10]. The most compelling utility of OoC systems is their capacity to replicate drug-specific Absorption, Distribution, Metabolism, and Excretion (ADME) profiles in human-relevant contexts. Liver-on-chip systems based on human primary hepatocytes or hepatocyte-like cells formed by induced pluripotent stem cells (iPSCs) can be used to assess drug-induced liver injury (DILI), which is a leading cause of late-stage clinical trial attrition [10,47]. These chips can mimic hepatic metabolism, cytochrome enzyme activity, and biliary clearance pathways, offering predictive insights into hepatotoxicity, bioavailability, and drug–drug interactions with precision that is better than that of animal models. Critically, OoC systems allow the incorporation of patient-specific or genetically varied human cells, which allows the study of individual variability in drug responses [48,49]. This ability is especially useful in the development of personalized medicine and in enabling the modelling of rare genetic diseases, ethnically diverse populations, and unique drug responses, which may not be available with homogeneous cell lines or nonhuman models. In a blinded, protein-binding-corrected analysis of 27 drugs across two donors, the Emulate Liver-Chip showed 87% sensitivity and 100% specificity for predicting drug-induced liver injury (DILI) [47]. The chip detected nearly 7 out of 8 drugs associated with clinical hepatotoxicity, despite prior animal testing. Figure 7 illustrates advanced organ-on-chip platforms and their comparative predictive performances [10,47].

#### 5.1.1. Case Study 3: Onpattro (Patisiran)—First FDA-Approved RNA Interference Therapeutic

Onpattro (patisiran), the first FDA-approved RNA interference (RNAi) therapeutic (2018) based on OoC testing with a nanotechnology-enabled delivery method, is an example of a drug used to treat hereditary transthyretin-mediated (hATTR) amyloidosis [50]. In preclinical development, liver-on-chip systems have been used to predict the hepatocyte uptake and metabolism of lipid nanoparticle (LNP) formulations. The LNP encapsulates siRNA in an approximately 80 nm liposomal vesicle that contains ionizable lipids, cholesterol, and PEG lipids, which shield the siRNA and allow it to be delivered to hepatocytes via apolipoprotein E-mediated endocytosis. Phase 3 clinical trials have indicated an 81% decrease in serum TTR protein levels and a notable decrease in neuropathy scores [50]. LNP technology tested by Onpattro subsequently allowed the rapid production of mRNA vaccines against COVID-19 (Pfizer-BioNTech, Moderna), demonstrating how preclinical OoC nanotechnology integration accelerates broad translational impact [51].

#### 5.1.2. Case Study 4: Recent Advances in AI-Enabled Drug Discovery

In recent years, AI-driven drug discovery has rapidly matured beyond isolated demonstrations of its potential. In 2022, Insilico Medicine progressed INS018_055 (rentosertib), an AI-designed TNIK kinase inhibitor for idiopathic pulmonary fibrosis (IPF), into Phase I clinical trials approximately 30 months after AI-driven target identification [52]. Phase I results demonstrated the safety and tolerability of this drug in healthy volunteers. Initial Phase IIa top-line results reported a dose-dependent FVC improvement in with IPF (NCT05938920) [18]. The previous results subsequently confirmed a mean FVC change of +98.4 mL in the 60 mg once-daily group compared with −20.3 mL in the placebo group [53]. Building on these results, Insilico Medicine initiated a Phase III trial of rentosertib (NCT07687459), a randomized, double-blind, placebo-controlled study patients across centers in China, making it the first fully AI-designed molecule targeting an AI-identified target to reach late-stage Phase III testing [54]. As this update is based on a sponsor announcement and clinical trial registry entry rather than a peer-reviewed publication, it should be interpreted cautiously, pending formal clinical results. Exscientia similarly advanced EXS21546, an AI-designed adenosine A2A receptor antagonist for solid tumors, through Phase 1a in 2022 and Phase 1b/2 trials in patients with RCC/NSCLC [19]. Beyond small molecules, AlphaFold 2 predicted the structures of more than 200 million proteins with near-atomic accuracy [55,56], and AlphaFold 3 (2024) extended this to protein–DNA, protein–RNA, and protein–ligand interactions directly relevant to drug discovery [57]. These examples demonstrate that AI drug discovery is progressing beyond isolated demonstrations to larger pipelines with clinical implications in mind. The contrast between baricitinib, an AI-assisted repurposed drug that has received FDA approval, and DSP-1181, an AI-designed novel molecule that did not advance beyond Phase I, illustrates that regulatory translation remains faster for repurposed compounds with established safety profiles than for novel ones.

### 5.2. Reduction in Animal Use and the 3Rs Principle

The integration of synthetic organ systems represents an enormous ethical and scientific advancement in preclinical studies. Traditional preclinical research relies on mice, rats, rabbits, and non-human primates to model human physiological and pathological conditions. However, preclinical outcomes are misleading because of genetic dissimilarities, metabolic differences, and divergent immune and drug response patterns between animal species and humans; drugs that seem safe and effective in animals are dangerous or useless in humans, with frequencies that lead to ~90% of clinical trial failures [4].

Artificial organ systems contribute to the internationally recognized 3Rs principle of animal research ethics [58].

- Replacement: Substituting animal models with other techniques, including in vitro assays, computational models, and organ-on-a-chip technologies.
- Reduction: Decreasing the number of experimental animals required by adopting superior research systems.
- Refinement: To alleviate animal suffering and improve animal welfare in cases where animals must be used.

Artificial organ systems offer significant economic and practical advantages. These systems facilitate drug testing at early stages of development, toxicology analysis, and experimentation of diseases at a more precise scale than possible with animal-based research [47], while simultaneously decreasing expenses and speeding up testing duration. Table 2 compares the main features of traditional animal models and artificial organ-on-chip systems used in preclinical studies.

There is increasing interest among regulatory bodies, such as the FDA, EMA, and OECD, in the adoption of organ-on-chip platforms for regulatory decision-making. Various groups are striving to develop standard protocols, validation studies and guidelines. In 2024, the Emulate Liver-Chip became eligible for the ISTAND qualification program of the FDA, with a sensitivity and specificity of 87% and 100%, respectively, for 27 blinded drugs. This paradigm shift in safety testing exemplifies the wider trends in biomedical innovation [10,47,59,60]. In April 2025, the FDA issued landmark guidance to phase out animal testing requirements for drug approval over a 3–5-year timeline, replacing them with new approach methodologies, including OoC systems and organoids [61]. The NIH simultaneously established the Office of Research Innovation, Validation, and Application (ORIVA) to accelerate the development and adoption of human-centric OoC technologies [61]. This represents the most significant regulatory shift in preclinical testing in the coming decades. Organ-on-chip technology has shown promising evidence of improved predictive performance over animal models, particularly in the Emulate Liver-Chip DILI study. However, this evidence comes from a single company-led study and has not yet been independently replicated across all platforms. Most OoC applications beyond DILI prediction remain in the proof-of-concept stage, while regulatory qualification represents an important step toward pharmaceutical adoption, but not yet routine.

## 6. Nanoscience and Nanotechnology: Advancing Drug Delivery at the Molecular Scale

Nanoscience is an interdisciplinary field that examines the physicochemical and biological properties of materials at the molecular level (1–100 nm). At this size, quantum confinement, surface effects, and enhanced reactivity become important for the behavior of the material, resulting in properties that are no longer similar to those of the bulk materials [62,63]. Nanotechnology uses nanoscale principles to design and fabricate functional objects by manipulating single molecules and atoms to produce new functional materials, systems, and devices with superior mechanical, optical, thermal, magnetic, and electrical properties.

**Figure 8:**
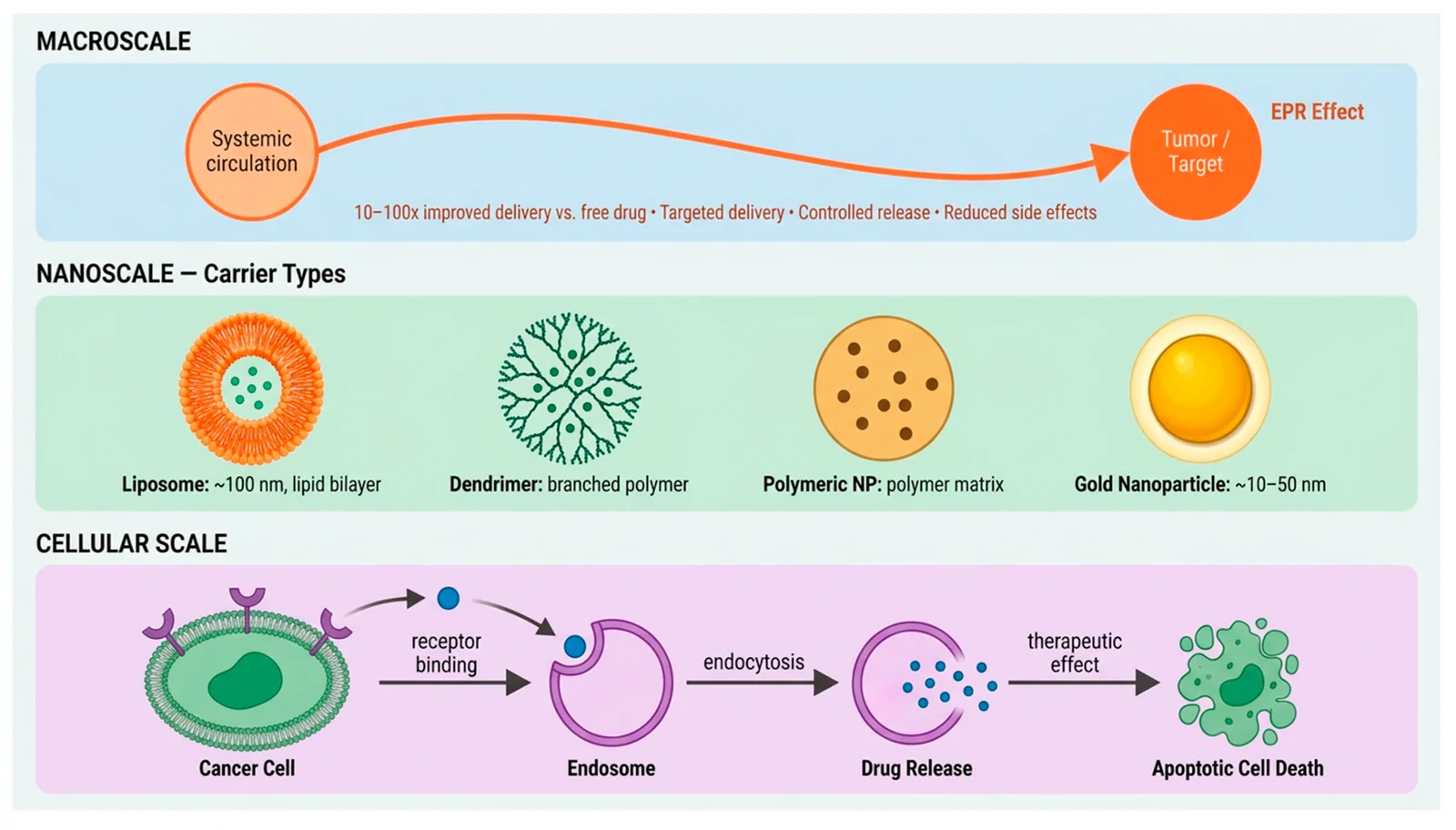
Multiscale nanoparticle-based drug delivery illustrates systemic circulation and tumor accumulation, nanocarrier architectures, cellular uptake, intracellular drug release, and therapeutic effects. Representative nanocarriers include liposomes, dendrimers, polymeric nanoparticles, and Au nanoparticles.

Nanomaterials are classified according to their dimensionality (0D, 1D, 2D, and 3D) and composition (organic, inorganic, and hybrid) [62]. The most prevalent pharmaceutical nanomaterials include carbon-based nanostructures (carbon nanotubes, fullerenes, and graphene), metal and metal oxide nanoparticles (silver, gold, zinc oxide, and titanium dioxide), quantum dots, lipid-based nanocarriers (liposomes and solid lipid nanoparticles), and polymeric nanoparticles. These multifunctional nanomaterials enable both standalone therapeutic applications and functional integration into complex drug delivery systems, resulting in technological advances in the pharmaceutical industry (Table 3). Medical nanotechnology has led to major advances in diagnostics, imaging, targeted therapies, regenerative medicine, and drug delivery. Engineered nanoparticles can be directed toward specific cancer cells, tissues, and cell populations to deliver therapeutic agents with reduced systemic toxicity and side effects. Nanoparticle-based drug delivery systems achieve 10–100-fold improvements in delivery efficiency compared to free drug administration, with benefits such as targeted delivery, controlled release, and reduced systemic side effects [9,64]. Figure 8 presents a multiscale visualization of nanoparticle-based drug delivery systems [9]. Long-term safety and reproducibility are also important challenges.

### 6.1. Nanotechnology in Drug Delivery: Clinical Validation

Nanoparticle-based drug delivery has achieved clinical success in multiple oncology and genetic disease indications, validating the translational potential of this platform [50,64,66,67].

- Doxil^®^ (liposomal doxorubicin, 1995): The first FDA-approved nanomedicine that delivers doxorubicin via PEGylated liposomes (~100 nm), enabling tumor accumulation via the enhanced permeability and retention (EPR) effect while dramatically reducing cardiotoxicity compared to free doxorubicin.
- Abraxane^®^ (albumin-bound paclitaxel, 2005): Nanoparticle albumin-bound (nab) paclitaxel (~130 nm) achieved an objective response rate of 33% compared with 19% for solvent-based paclitaxel in metastatic breast cancer (~74% relative increase).
- Genexol-PM^®^ (paclitaxel in mPEG-PLA micelles): A polymeric micellar formulation approved for metastatic breast cancer that eliminates the toxic Cremophor EL solvent required for the conventional paclitaxel formulation.
- Onpattro^®^ (patisiran LNP-siRNA, 2018): As detailed above, the first RNAi therapeutic demonstrated an 81% TTR reduction in hATTR amyloidosis using lipid nanoparticle delivery and validated the LNP platform subsequently used in mRNA COVID-19 vaccines.

The LNP platform validated by Onpattro has rapidly evolved since its inception. Novel ionizable lipids and selective organ targeting strategies now enable tissue-specific mRNA delivery beyond the liver, including to the lungs, spleen, and tumor microenvironment [68]. Emerging RNA modalities, including self-amplifying RNA and circular RNA, combined with next-generation biodegradable LNPs, aim to improve therapeutic durability and expand nanomedicine into oncology, autoimmunity, and rare genetic diseases [69]. All these approvals point to one thing in common: nanoformulation is a method for solving the basic problem of delivering fragile or poorly soluble drugs to their targets with acceptable safety and efficacy profiles. The degradation of free siRNA, such as that of serum nucleases, which occurs within minutes, is reduced by LNP-siRNA encapsulation, producing sustained therapeutic effects with monthly dosing.

### 6.2. Physicochemical Properties of Nanoparticles in Medicine

**Figure 9:**
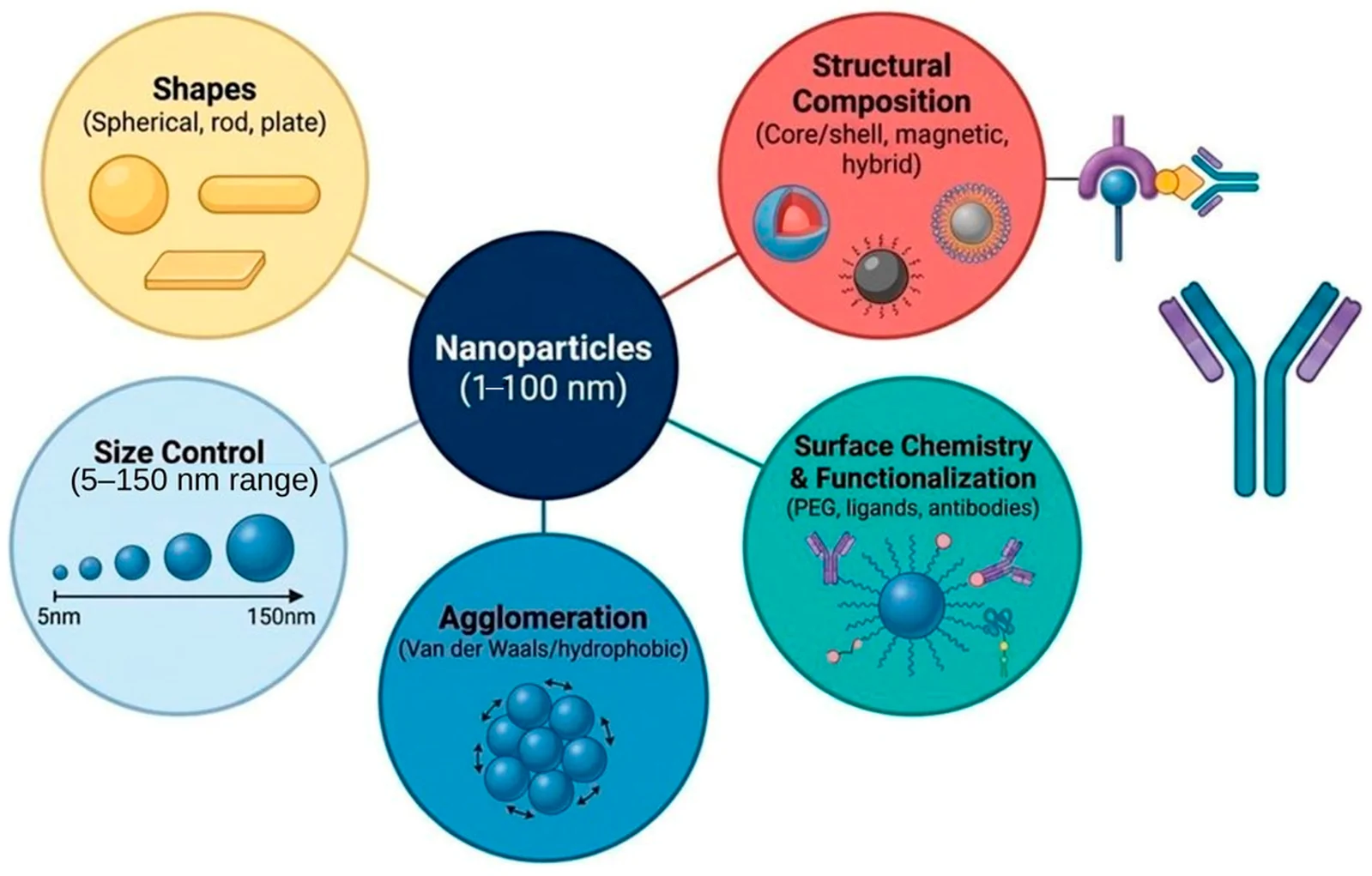
The physicochemical properties of nanoparticles, including shape, composition, surface chemistry, agglomeration, and size control, are relevant to their behavior and performance in biomedical applications.

Systems based on nanoparticles are controlled by a constellation of physicochemical properties that can be systematically designed to dictate their therapeutic performance. These properties are shown in Figure 9 [63].

#### 6.2.1. Nanoscale Dimensions and Enhanced Pharmacological Behavior

Nanoparticles in the 1–100 nm size range have high surface-to-volume ratios, enabling increased contact with biological environments and improved cellular absorption and penetration of physiological barriers (e.g., blood–brain barrier to CNS-targeted therapies). Passive tumor accumulation using EPR is size-dependent and is optimized at 50–200 nm [9].

#### 6.2.2. Tailorable Physical and Chemical Characteristics

The size, shape, surface area, and surface chemistry of nanoparticles can be systematically adjusted to maximize drug loading/release patterns, circulation times and biodistribution, cellular uptake and targeting, and immune evasion properties [63].

#### 6.2.3. Types of Nanoparticles and Their Biomedical Applications [62,63,64,65]

- Liposomes: Lipid bilayer vesicles that can carry hydrophilic drugs in the aqueous core and hydrophobic drugs in the bilayer. Examples include Doxil, Onpattro, and messenger RNA (mRNA) COVID-19 vaccines.
- Polymeric Nanoparticles (~50–300 nm): PLGA, PLA, chitosan, and PEG-based nanoparticles with controlled and tunable drug release. These include Eligard (leuprolide) and Abraxane (albumin-based nanoparticles).
- Dendrimers (~1–10 nm): Branched synthetic polymers with monodisperse size, high drug-loading capacity, and multivalency for targeted cancer therapy and gene delivery applications.
- Inorganic Nanoparticles: Gold nanoparticles in photothermal therapy and imaging; iron oxide in MRI contrast and magnetic targeting; silica in controlled release; and quantum dots in diagnostics (characterization of toxicity is ongoing).
- Gold Nanoshells: Utilized in photothermal therapy via near-infrared light absorption.
- Polymeric Micelles: Solubilize poorly water-soluble drugs and enhance their bioavailability.

#### 6.2.4. Common Synthesis Methods

Synthesis techniques that can be used to generate nanoparticles include emulsification, nanoprecipitation, solvent evaporation, and microfluidic techniques. These methods offer accurate control over the size, shape, and surface characteristics of the nanostructures. Synthesis using microfluidics is becoming a standard method of manufacturing that is GMP-compliant and reproducible [63].

#### 6.2.5. Surface Chemistry, Functionalization, and Targeting

Nanoparticles can be functionalized with targeting ligands (antibodies, peptides, and aptamers), hydrophilic polymers (e.g., PEG to promote longer circulation), and imaging agents. Active tumor targeting through the conjugation of tumor-specific antibodies further enhances tumor selectivity over passive accumulation via the EPR effect [9].

#### 6.2.6. Stimuli-Responsive and Theranostic Systems

Advanced nanoparticles can be designed for simultaneous therapy and diagnostics (theranostics), dual and multi-drug delivery, and stimuli-responsive release systems based on pH, temperature, redox potential, and enzyme activity [70]. These systems represent the future of nanomedicine.

#### 6.2.7. Biological Reactivity and Safety Considerations

While greater surface energy and reactivity can increase the rate of drug delivery, they can also augment toxicity risks, either by oxidative stress, complement activation, or unintentional accumulation in non-target organs. The bioavailability and cytotoxicity profiles of nanoparticles can be modified through agglomeration, which is caused by van der Waals or hydrophobic interactions. PEGylation and other surface coating methods are commonly used to avoid aggregation and prolong the circulation of NPs in the bloodstream of the host. To ensure safety, comprehensive toxicological testing, including long-term toxicity and immunogenicity assessments, is required [63]. Beyond the acute effects, nanotoxicology examines long-term outcomes, such as organ accumulation, chronic inflammation, and genotoxicity, which remain incompletely characterized for many newer nanocarrier chemistries. Manufacturing scale-up also presents GMP challenges, including batch-to-batch variability in particle size and drug loading. Although the enhanced permeability and retention (EPR) effect is well-established in preclinical tumor models, its substantial heterogeneity across human tumors and patients limits its reliability as a universal basis for passive targeting [63,64]. Nanotechnology has greater clinical maturity than organ-on-chip and AI/ML platforms, as evidenced by multiple approved nanomedicines. However, clinical translation remains concentrated in established formulations, such as liposomal and albumin-bound therapeutics, while multifunctional and actively targeted nanoparticles remain largely preclinical.

## 7. Technology Integration and Synergies

As shown in Table 4, emerging drug discovery technologies, such as AI/ML, pharmacogenomics, organ-on-chip systems, and nanotechnology, have significant potential to accelerate drug development and improve therapeutic precision. Among these, pharmacogenomics and nanotechnology exhibit relatively higher clinical and regulatory maturity, whereas AI/ML and organ-on-chip technologies are still evolving in terms of validation and large-scale clinical translation. Despite their advantages, challenges such as high costs, scalability limitations, data bias, regulatory uncertainty, and long-term safety concerns continue to hinder widespread implementation.

**Figure 10:**
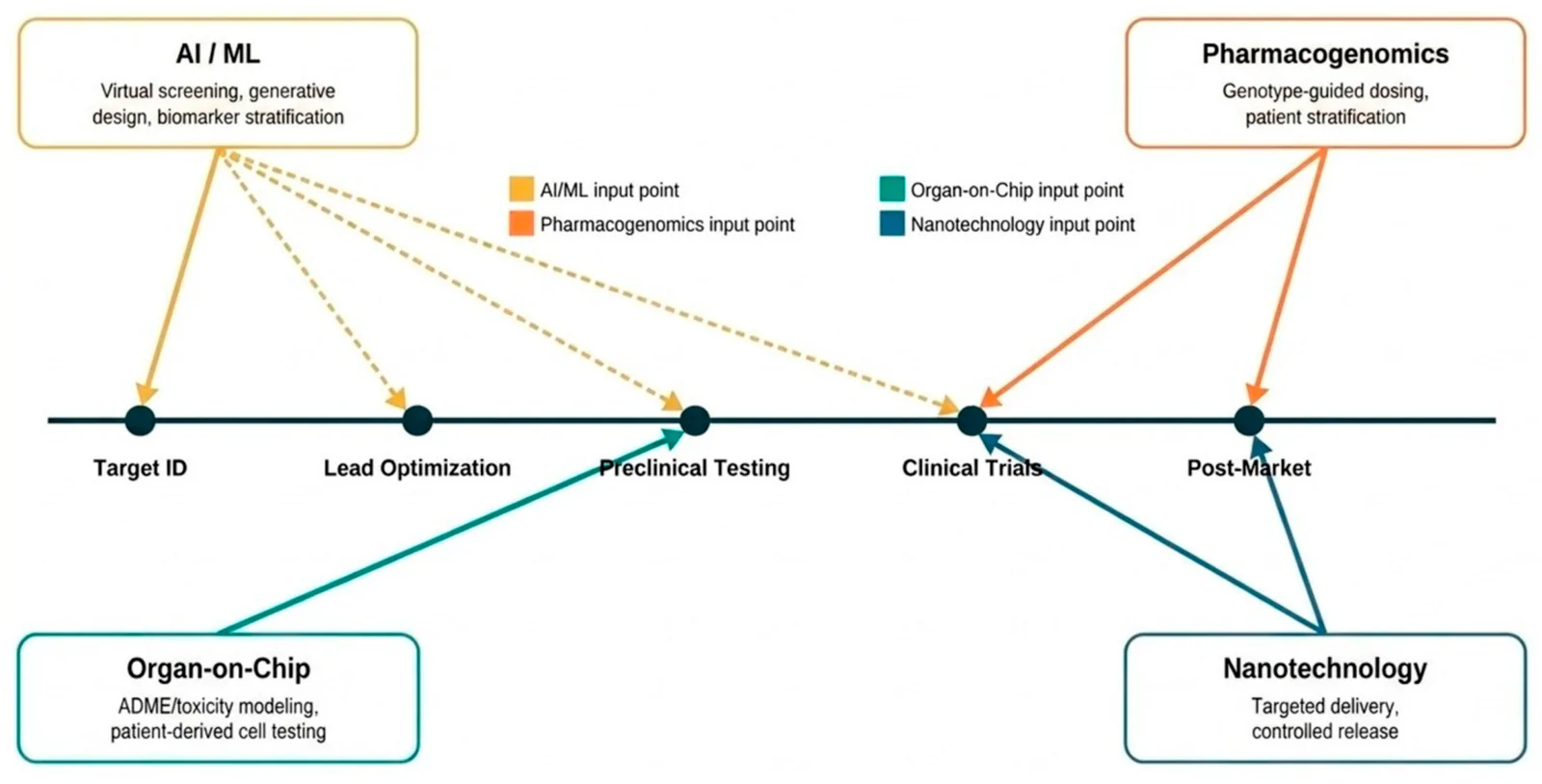
Technology interaction across the drug discovery pipeline. Schematic showing AI/ML, organ-on-chip, pharmacogenomics, and nanotechnology as inputs feeding successive stages of drug discovery and development, from target identification through post-market evaluation.

Although this table provides a systematic comparison, important context is required. AI/ML clinical translation is uneven: baricitinib achieved full FDA approval in May 2022, while DSP-1181 did not advance beyond Phase I and halicin remains preclinical. Pharmacogenomics is the most mature technology and has already been integrated into routine clinical practice, although equity and privacy issues remain. Organ-on-chip systems offer high potential but face challenges in terms of their reproducibility and regulatory validation. Examples of nanotechnology, such as Onpattro, demonstrate that complex nanomedicines can achieve approval, although manufacturing scalability and long-term safety monitoring remain active challenges in this field. It is not a single application of AI, organ-on-chip technology, nanotechnology, or personalized medicine, but a strategic combination of these areas that will lead to transformative potential. Figure 10 illustrates how AI/ML, pharmacogenomics, organ-on-chip systems, and nanotechnology interact at specific stages of the drug discovery pipeline target identification, lead optimization, preclinical testing, clinical trials, and post-market evaluation, providing the structural basis for the timeline comparison that follows.

**Figure 11:**
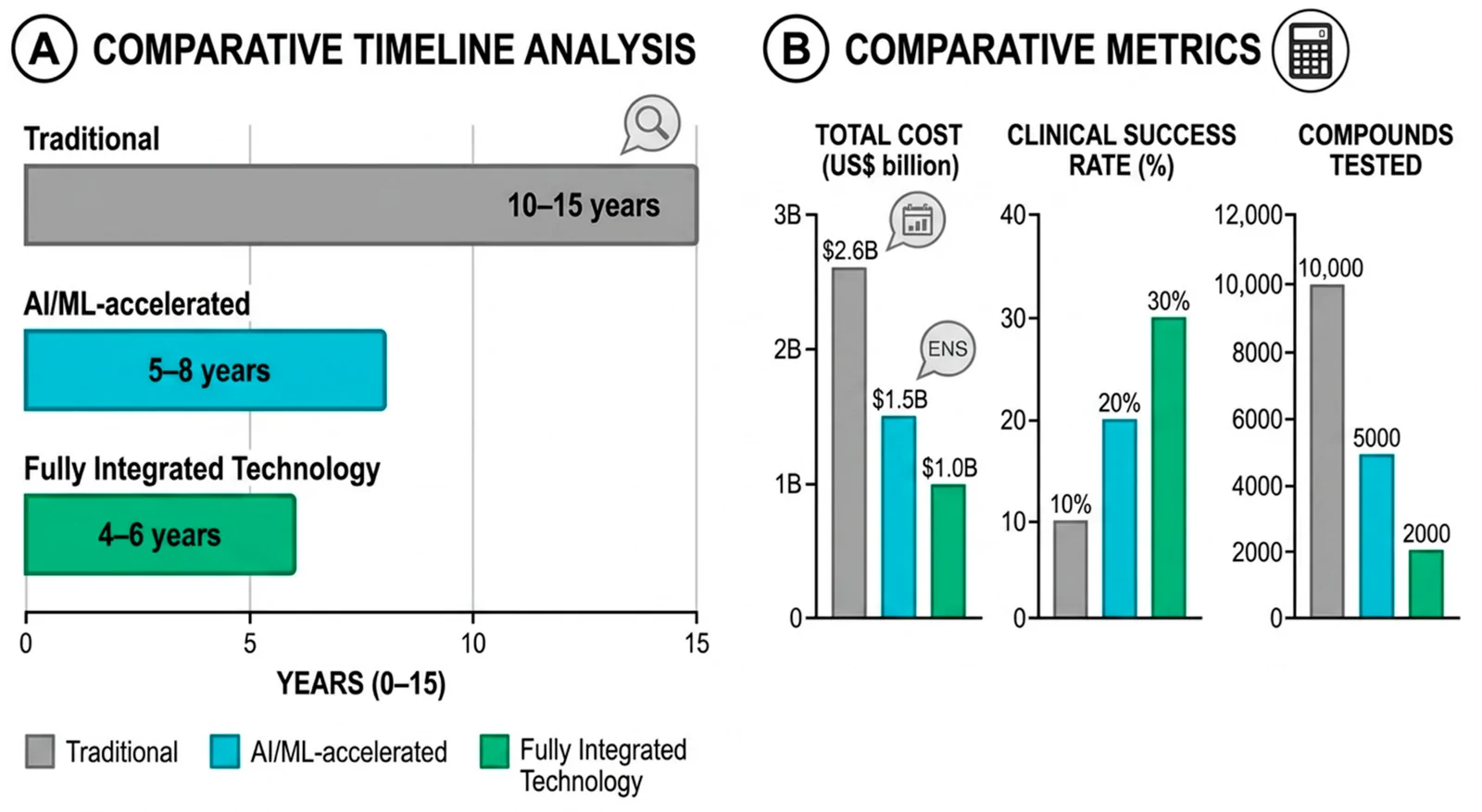
Projected comparison of traditional, AI/ML-accelerated, and fully integrated drug development pathways in terms of development time, cost, and projected success rates.

These technologies focus on different bottlenecks in the drug development pipeline, and their combination results in a synergistic effect that is greater than the sum of their parts [7,10]. This convergent effect is quantified in Figure 11 (Comparative Timeline Analysis), which presents the modelled/projected estimates based on the published data. Development time is projected to decrease from the traditional range of 10–15 years to 4–6 years with a fully integrated technology pathway, while the total cost is projected to decrease from approximately $2.6 billion to $1.0 billion. Estimates are based on the published literature [1,2,4]. The clinical success rate is projected to increase from approximately 10% to 30% (3-fold), and the number of compounds requiring physical testing is projected to decrease from 10,000 to 2000 (80% reduction) [1,2,4].

### 7.1. AI and Organ-on-Chip Integration

The integration of AI and OoC forms a potent loop of iterative feedback: AI predicts drug candidates → organ chips prove predictions correct → experiments improve AI models → better predictions in the next cycle. This loop was used to further develop the computational and experimental elements in this study. AI–OoC integration creates iterative feedback loops, whereby computational predictions are refined using experimental chip data to progressively improve model accuracy. However, specific performance projections vary considerably by platform and have not yet been independently validated. The reproducibility of chip models and limited regulatory acceptance remain key challenges for their widespread adoption [10,47]. However, these figures are platform-dependent and have not yet been validated universally across independent studies.

### 7.2. Nanotechnology and Personalized Medicine Convergence

Individual nanoformulations are a merger of genomics and material science. Patient genetic profiling determines the most effective drug combinations and dosing requirements, and nanotechnology allows the simultaneous delivery of multiple therapeutic factors with specific and regulated release properties. Pharmacogenomic information can be used to design nanoparticles: depending on the metabolic enzyme variant of the individual patient, a modified drug loading or release profile may be required to attain therapeutic plasma concentrations without toxicity [9,29].

### 7.3. AI-Driven Patient Stratification in Clinical Trials

The design of clinical trials is being changed by AI-based patient stratification using biomarker analysis. Instead of drugs being tested in diverse populations, AI can identify genomically defined subgroups with the highest probability of response, allowing smaller, quicker, and more powerful trials to be conducted. AI-driven biomarker stratification has demonstrated improved response rates in oncology trials and has enabled smaller and faster studies [32]. Although specific figures vary considerably across cancer types and trial designs, genomically enriched populations consistently improve the statistical power and reduce the required sample size. However, their generalizability to broader populations may be limited by the availability of biomarkers and diversity of training data [32]. The integration scenarios discussed in this section (Figure 10 and Figure 11) represent the most speculative aspects of this study. Although individual technology combinations, including AI–OoC feedback, nanoparticle–pharmacogenomic co-design, and AI-based trial stratification, have some empirical support, the timeline and cost estimates in Figure 11 are model projections rather than observed outcomes from an integrated program. Therefore, they should be interpreted as illustrative scenarios rather than forecasts.

## 8. Ethical Considerations in AI- and Technology-Driven Drug Discovery

The combination of AI and ML in drug discovery is transforming the pharmaceutical industry by allowing the identification of drug candidates more quickly, accurately, and at a lower cost [5,7]. However, alongside these advances, large ethical issues must be actively addressed to provide responsible and fair innovations (Table 5).

### 8.1. Key Ethical Concerns [71,72,73]

- Data Privacy and Consent: The processing of sensitive patient data to train AI models requires strong data anonymization, informed consent, and adherence to legal requirements, such as the GDPR and Health Insurance Portability and HIPAA.
- Algorithmic Bias and Fairness: Algorithms trained on non-representative data reproduce biases, which may result in disproportionate drug efficacy and safety predictions depending on the racial, gender, and socioeconomic groups. The results of historical clinical trials have disproportionately represented certain demographics, reducing the generalizability of AI models.
- Transparency and Explainability: Predictions with complex DL systems have a black box quality that hides the logic behind the prediction and makes it challenging to be approved by regulators and clinically adopted. Explainable AI (XAI) algorithms, including feature importance scores, attention mechanisms, and counterfactual explanations, are currently being developed to address this challenge.
- Accountability and Responsibility: There need to be clear lines of accountability regarding AI-driven decisions that cause patient harm, including developers, pharmaceutical companies, regulatory bodies, and clinicians.
- Intellectual Property: Current legal frameworks are inadequately equipped to handle ownership rights for AI-generated discoveries and inventions.
- Equitable Access: The high cost of AI technologies risks widening global health disparities, particularly disadvantaging low- and middle-income countries.
- Malicious Use: The potential misuse of AI in designing biochemical threats underscores the need for strict oversight and international ethical safeguards.

### 8.2. Proposed Solutions and Evolving Regulatory Frameworks [72,74,75]

- Regulatory Guidance: The FDA’s AI/ML-Based Software as a Medical Device (SaMD) Action Plan (2021) and the EMA’s Reflection Paper on Artificial Intelligence in the Medicinal Product Lifecycle (2022) are important initial steps towards well-defined routes for the validation and monitoring of AI tools in the healthcare sector.
- Technical Solutions: XAI techniques, federated learning (enabling model training and training across several institutions without exchanging raw patient data), and model fact sheets will be developed to increase transparency and apply bias auditing prior to implementation.
- Operational Practices: Algorithmic impact assessments prior to deployment, pre-specified performance benchmarks for diverse demographic subgroups, and post-deployment surveillance with continuous model retraining should be implemented.

## 9. Challenge and Limitations

Despite these impressive advancements, the effects of these technologies are limited by serious technical, operational, and systemic problems. Realistic adoption planning requires a balanced critical appraisal.

### 9.1. AI and ML Limitations

The validation rates are relatively low; the AI-predicted compounds that pass wet-lab validation tests represent only a fraction of those that subsequently enter clinical trials, and the validation rates vary considerably by platform and compound class [76]. The lack of explainability of the safety-critical decisions presented by DL models makes their use uncertain, and their use has regulatory uncertainty because of their black box nature, which cannot be frequently explained by current architectures [23]. Concerns persist regarding the reproducibility of AI-driven drug discovery models, stemming in part from inadequate reporting of methods and proprietary or inconsistent training data [76]. Standardized benchmarks (e.g., MoleculeNet) can be useful, although differences between training datasets and real-world applications remain an issue, particularly when models are trained on data from high-income populations [76]. Multi-objective optimization (MOO) is a persistent challenge, with most AI applications optimizing conflicting goals (e.g., potency, selectivity, and metabolic stability). The development of effective algorithms that explore such trade-offs is an active area of research. Catastrophic forgetting, where neural networks forget information learned during the learning of new tasks, also makes it more difficult to build continuously improving AI systems to discover drugs [77].

### 9.2. Organ-on-Chip Challenges

Existing OoC systems cannot replicate organ complexity. Most chips do not have built-in immune system components, multi-organ interactions (with the exception of body-on-a-chip systems), or long-term culture stability beyond days to weeks. Scalability is a constraint on throughput: the cost per chip of $50–500 means that it is used in late-stage candidate validation and not in early-stage screening [10]. Standardization is still lacking in cross-platform applications and is not accepted by regulatory bodies, except for a few FDA-qualified models. Special expertise is required to address technical issues, such as maintaining physiological flow rates, avoiding the formation of bubbles in microchannels, and reproducible cell seeding, which restricts their use to research facilities with available funding [46].

### 9.3. Nanotechnology Manufacturing and Safety Concerns

The manufacture of nanoparticle therapeutics faces challenges, such as variations in batch-to-batch size distribution, drug loading, and surface properties. Reformulation may also be necessary to scale from the bench to GMP production, thus contributing to increased development time and costs [63]. Currently, long-term safety data for many novel nanoformulations are unavailable. Although acute safety is thoroughly described with approved drugs such as Doxil and Onpattro, there is a lack of 10–20-year safety data. The accumulation of nanoparticles in the liver, spleen, and other organs raises questions regarding their chronic toxicity. Regulatory routes for novel multifunctional nanoparticles (to perform targeting, imaging, and therapeutic functions) have not yet been clearly established [64].

### 9.4. Personalized Medicine Barriers

Many diseases still lack validated predictive biomarkers that can reliably guide therapy selection, limiting the broader implementation of precision medicine. Polygenic interactions are genetically complex and cannot be studied using single gene methods. Economic obstacles are high; the overall cost of genomic profiling is between $1000–$5000 per patient, and developing a companion diagnostic is an extra $50–$100M to the cost of development. The issues of equity in healthcare are urgent because the infrastructure of personalized medicine is concentrated in high-income nations, which may contribute to increasing healthcare disparities. Genomic profiling (1–3 weeks) is known to have a slow turnaround, which can delay the initiation of treatment for aggressive diseases; thus, point-of-care testing innovations must be rapid.

### 9.5. Vascularization in Bioprinted Constructs

The microfluidic system and layer-by-layer assembly of tissue engineering have not completely addressed the issue of creating thick microtissue constructs with sufficient vasculature. Hypoxic conditions are a major problem for vascularization, killing cells by apoptosis in constructs more than 200 μm thick [78]. This inherent shortcoming can be addressed by developing sufficient vascularization strategies, such as vascular network bioprinting, incorporation of proangiogenic factors, and sacrificial templating, before scaling bioprinted tissues to clinical applications. The key technical and ethical challenges facing AI adoption in drug discovery, including data-related limitations, multi-objective optimization, reproducibility, confounding variables, model appropriateness, catastrophic forgetting, terminology inconsistency across the field, and organizational adoption barriers, are discussed throughout this section and summarized in the limitations section [5,7,71].

## 10. Future Perspectives

The innovations discussed in this review have the potential to support more efficient, accurate, and patient-centered drug development. Among these, the combination of AI-accelerated drug repurposing with human-relevant OoC testing represents a promising strategy for future investigations. The high costs and long development times mentioned in the introduction are directly addressed by this convergence through the identification of safe, pre-existing compounds for novel diseases and testing them in predictive human models, which could help reduce early-stage development risks and potentially decrease attrition before clinical testing.

### 10.1. Advancing Drug Discovery and Development

AI, especially DL models, such as transformer-based models, such as the variants of GPT used with molecular design, are actively being introduced into regular discovery processes. AI models are likely to become increasingly integrated into drug discovery workflows, although their ability to improve clinical success rates requires further validation. The eventual approval of a drug discovered substantially with AI assistance could provide important evidence regarding the potential economic impact of AI-enabled drug development. AI-informed candidate selection may improve early clinical development outcomes; however, evidence remains limited and is undergoing prospective evaluation on a larger scale [4].

### 10.2. AI Integration in Pharma R&D

The use of AI in pharmaceutical R&D is expected to continue to grow. Industry surveys suggest that while AI adoption is widespread among pharmaceutical companies, translation into fully integrated pipeline applications remains less common [79]. The deepening partnership between AI-native biotech firms and major pharmaceutical companies, as reported in company announcements by the Sanofi–Exscientia (up to $5.2B milestone-based deal, 2022), Roche–Recursion ($150M, 2023), and AstraZeneca–BenevolentAI partnerships, illustrates the growing collaboration between AI-focused biotechnology companies and major pharmaceutical organizations. Industry analysts project substantial continued growth in the AI drug discovery market through the end of the decade, although estimates vary by source and methodology.

### 10.3. Transforming Cancer Medicine with AI and Precision Oncology

AI-driven cancer care has the potential to support more accurate and personalized treatment through the integration of multi-omics data, explainable AI, federated learning, and individualized patient profiling. AI-directed biomarker selection combined with OoC validation may improve predictive performance in precision oncology; however, the magnitude of this improvement requires validation in independent studies and clinical settings [32].

### 10.4. Toward a New Era of Precision Medicine

The combination of AI, pharmacogenomics, OoC testing, and nanotechnology-based delivery represents an important future direction in precision medicine. The convergence of these technologies could improve treatment personalization and potentially benefit patient outcomes, although their effects on survival, health inequalities, and QoL require clinical validation [80]. Fairness, transparency, and social accountability in AI implementation, as well as ethical and responsible AI, are crucial to ensure that these advancements benefit all populations equally.

### 10.5. Addressing Challenges for Broader Technology Adoption

To realize the potential of these technologies, several critical obstacles must be systematically addressed: low-quality data and restricted access; biases in algorithms to produce population-wide outcomes; inconsistencies in regulations across geographic borders; low patient engagement and confidence in AI systems; and a lack of infrastructure in low- and middle-income countries [5,72]. To facilitate ethical and collaborative development, interdisciplinary collaboration between computational scientists, experimental researchers, clinicians, regulatory scientists, and patient advocates is required.

## 11. Scope and Limitations

This review has some limitations. First, it is a narrative rather than a systematic review; although a structured search strategy was applied (Section 2), the study selection and synthesis involved subjective judgment rather than the standardized and reproducible procedures used in systematic reviews and meta-analyses. Second, some recent clinical-stage claims, particularly the initiation of the Rentosertib Phase III trial, rely on sponsor communications and clinical trial registry information because peer-reviewed data are not yet available; therefore, these findings should be interpreted cautiously pending formal publication. Third, the five technology domains reviewed differ substantially in terms of evidence maturity and volume, resulting in varying depths of critical analysis. Finally, several headline statistics, including organ-on-chip predictive performance and projected AI-driven reductions in development time and cost, are based on individual studies or industry-reported/modelled estimates rather than independently replicated evidence and should, therefore, be considered illustrative rather than generalizable.

## 12. Conclusions

The combination of AI/ML, 3D bioprinting, organ-on-chip technology, and nanotechnology can be considered a complete set of tools to directly address the dual problems of high costs and long development times, which have traditionally limited pharmaceutical R&D. Not future ideas but current engines of pharmaceutical development are illustrated through real-world case studies, such as the AI-driven discovery of baricitinib against COVID-19, the first AI-designed clinical candidate DSP-1181, and the nanotechnology-based breakthrough of Onpattro.

AI and ML have powerful quantitative frameworks that simplify all stages of workflows, such as pre-target identification and adaptive clinical trial designs. Simultaneously, 3D bioprinting and organ-on-chip technology are solving the long-standing predictive failure of conventional preclinical models, closing the translational gap between animal studies and human trials, and lowering the high rates of attrition, which significantly contributes to the development costs of new drugs. The FDA qualification of several OoC systems is a milestone in regulatory science and an indicator of the institutional acceptance of these technologies.

Nanotechnology-enabled drug delivery, which has been proven successful by numerous approved nanomedicines in oncology and genetic diseases, has opened the therapeutic frontier to drugs that were previously thought to be inapplicable. Pharmacogenomics and personalized medicine offer broad clinical strategies, according to which the application of these technologies in relation to specific patients can be defined.

In the future, the combination of AI and advanced bioengineering platforms will be the leading approach. This synergy will allow the optimization of multi-organ systems, decipher the complex pathology of diseases, and eventually provide maximally effective and highly personalized treatments for patients with diabetes. Close cooperation between industry, academia, and regulatory bodies will be pivotal for the standardization and exploitation of these technologies in the future. Convergent technologies are eventually converging to recreate human biology with an unprecedented precision never seen before to usher in a new age of biomedicine and a more sustainable and humane paradigm of global healthcare.

## Figures and Tables

**Table 1:** Representative Gene–Drug Pairs and Clinical Recommendations in Pharmacogenomics.

Gene	Drug(s)	Clinical Recommendation
*CYP2C19*	Clopidogrel	Poor/intermediate metabolizers: consider alternative antiplatelet therapy [35]
*VKORC1*/*CYP2C9*	Warfarin	Adjust starting dose based on genotype-informed dosing algorithm [36]
*TPMT*/*NUDT15*	Thiopurines (e.g., azathioprine)	Reduce starting dose or select alternative agent in poor metabolizers [34]
*CYP2D6*/*CYP2C19*	SSRIs/antidepressants	Adjust dose or select alternative agent based on metabolizer phenotype [37]
*UGT1A1*	Irinotecan	Reduce starting dose in UGT1A1*28 homozygotes [34]
*SLCO1B1*	Statins	Reduced-function allele carriers: use a lower starting dose or select an alternative statin to reduce myopathy risk [35]

**Table 2:** Comparison of Traditional Animal Models vs. Artificial Organs (organ-on-chip) in Preclinical Research [4,47,58,59].

Aspect	Traditional Animal Models	Artificial Organs (Organ-on-Chip)
Physiological relevance	Limited due to interspecies differences	High; mimics human-specific organ functions
Predictive accuracy	Often poor (50–60%); leads to failures in human trials	87% sensitivity and 100% specificity for DILI prediction across 27 blinded drugs in a single published study; this result has not yet been independently replicated across laboratories or platforms, and the broader OoC literature reports variable performance depending on platform and organ type.
Ethical concerns	High due to animal use and potential suffering	Minimal; adheres to 3Rs ethical research standards
Cost and time	High; involves animal housing, care, and long study durations ($10,000–$50,000 per compound)	Lower; faster testing and cost-effective setup ($50–$500 per chip)
Personalization potential	Not possible	Possible using patient-derived iPSC-derived cells
Regulatory acceptance	Established but under increasing ethical scrutiny	Emerging; FDA accepted the Emulate Liver-Chip into the ISTAND qualification programme (September 2024), with 87% sensitivity and 100% specificity across 27 blinded drugs
Reproducibility	Variable due to biological differences between animals	High; standardized microfluidic design and controlled conditions

**Table 3:** Potential Application Areas Where Nanotechnology Has a Significant Impact [9,62,65].

Area	Potential Applications
Healthcare	Development of personalized nanomedicines; precise and accurate drug delivery systems; nanoparticle-based diagnostics and theranostics; cochlear implants and retinal implants; nerve regeneration scaffolds.
Energy	Nanomaterial-enhanced batteries and supercapacitors can store large amounts of energy more efficiently and reduce fuel consumption. Stronger and lighter nanomaterial composites can be used to manufacture vehicles.
Environment	Pollutant removal with molecular sieves to trap contaminants, water, and air pollution with disposable nanosensors, and water purification using nanostructured membranes.
Electronics	Development of faster, smaller, and more efficient microprocessors, nanoscale transistors for miniaturizing devices, and flexible electronic circuits.
Consumer Goods	Nanotechnology in the cosmetic industry helps produce antimicrobial textiles, sports equipment, packaging materials, and titanium dioxide nanoparticles in sunscreens to offer better UV protection.

**Table 4:** Comparative Framework for Emerging Drug Discovery Technologies.

Technology/Case Study	Readiness Level	Regulatory Maturity	Scalability	Cost Implications	Key Limitations
AI/ML—Baricitinib	Repurposed drug; FDA-approved (May 2022)	Full approval achieved	Established manufacturing infrastructure for a repurposed small molecule	Discovery-stage costs reduced through virtual screening; low incremental cost for an existing drug	Repurposing success does not necessarily generalize to novel molecules
AI/ML—DSP-1181	Discontinued after Phase I	Did not reach approval	N/A	Discovery-stage savings realized; no downstream return	Rapid nomination did not prevent clinical failure
AI/ML—Halicin	Preclinical	Not yet in clinical development	Virtual screening scales readily; no clinical manufacturing yet	Discovery-stage savings only	No clinical validation to date
AI/ML—Rentosertib (INS018_055)	Phase III, initiated July 2026	Most advanced AI-designed novel molecule in clinical trials	Manufacturing scoped to a single-indication clinical program	Long-term costs remain uncertain during Phase III	Clinical outcome not yet established
AI/ML—EXS21546	Phase 1b/2	Early clinical stage	Manufacturing scoped to early-phase clinical supply	Ongoing clinical development costs	Efficacy remains to be established
AI/ML—AlphaFold 2/3	Structure-prediction tool; widely adopted	Not a therapeutic; not FDA-regulated	Computational tool; scales without manufacturing constraints	Low marginal cost per use	Predicts molecular structure, not clinical efficacy or safety
Pharmacogenomics	Clinical implementation	Established clinical guidelines	Scalability depends on genotyping infrastructure and lab capacity	Genotyping $1000–$5000/patient; infrastructure costs persist	Implementation varies across healthcare systems
Organ-on-Chip	Translational/early clinical adoption	Emerging regulatory qualification	Scalability is platform-dependent and currently limited by per-chip cost	May reduce animal-testing costs [47,58]; per-chip cost ($50–$500) currently limits throughput to late-stage validation rather than early screening [10]	Standardization and cross-platform validation remain limited
Nanotechnology	Multiple approved products; broader applications remain developing	Established for several nanomedicines	Scalability is constrained by GMP manufacturing scale-up	High production costs, particularly at GMP scale-up; potential long-term savings from improved therapeutic index have not been independently quantified across approved products	Long-term safety, reproducibility, and targeted-delivery challenges

**Table 5:** Ethical Principles of Healthcare and Medical Research Extended to Medical AI Applications [71,72,73].

Ethical Principle	Healthcare and Medical Research	Medical AI Application
Respect for Autonomy	Physicians and researchers respect individuals rights to make their own healthcare and research participation decisions.	Developers and users of AI must ensure that people have adequate control over their interactions with AI.
Beneficence	Medical staff operate for the good of the patient while upholding patients’ rights.	AI developers and users are responsible for maximizing human benefits and minimizing AI-related harm caused by AI.
Nonmaleficence	The medical team avoids both intentional and unintentional harm and negligence.	AI developers and users are responsible for preventing harm and actively mitigating risks during the design and deployment phases of AI systems.
Justice	Doctors and researchers deliver equal treatment without considering race, gender, socioeconomic background, or diagnosis.	AI developers and users should promote equity, regardless of race, gender, socioeconomic status or medical condition.
Accountability	Researchers and physicians are responsible for their actions and their outcomes.	It is the responsibility of AI developers and users to ensure that AI is designed, implemented, and managed ethically, transparently, and reliably.

## Data Availability

No new data were created or analyzed in this study. Data sharing is not applicable to this article.

## Abbreviations

The following abbreviations are used in this manuscript.

AI	Artificial Intelligence
ML	Machine Learning
DL	Deep Learning
ANN	Artificial Neural Network
HTVS	High-Throughput Virtual Screening
QSAR	Quantitative Structure–Activity Relationship
ADMET	Absorption, Distribution, Metabolism, Excretion, and Toxicity
ADME	Absorption, Distribution, Metabolism, and Excretion
3D	Three-Dimensional
FDA	Food and Drug Administration
EMA	European Medicines Agency
OECD	Organisation for Economic Co-operation and Development
MPS	Microphysiological Systems
OoC	Organ-on-Chip
CPIC	Clinical Pharmacogenetics Implementation Consortium
DPWG	Dutch Pharmacogenetics Working Group
iPSC	Induced Pluripotent Stem Cell
ECM	Extracellular Matrix
DILI	Drug-Induced Liver Injury
EPR	Enhanced Permeability and Retention
LNP	Lipid Nanoparticle
RNAi	RNA Interference
PEG	Polyethylene Glycol
PLGA	Poly(lactic-co-glycolic acid)
CYP450	Cytochrome P450
GDPR	General Data Protection Regulation
HIPAA	Health Insurance Portability and Accountability Act
XAI	Explainable AI
CAD	Computer-Aided Design
SLA	Stereolithography
FDM	Fused Deposition Modeling
DLP	Digital Light Processing
MOO	Multi-Objective Optimization
OCD	Obsessive–Compulsive Disorder
hATTR	Hereditary Transthyretin-Mediated Amyloidosis
TTR	Transthyretin
